# Case Series of Potential Cardiac Inflammation Associated With Various SARS-CoV-2 Vaccinations Assessed by Cardiac MRI

**DOI:** 10.3389/fcvm.2022.829392

**Published:** 2022-04-08

**Authors:** Constantin Jahnke, Patrick Doeblin, Radu Tanacli, Undine Witt, Matthias Schneider, Christian Stehning, Burkert Pieske, Sebastian Kelle

**Affiliations:** ^1^Department of Cardiology, German Heart Centre Berlin, Berlin, Germany; ^2^German Centre for Cardiovascular Research DZHK, Partner Site Berlin, Berlin, Germany; ^3^Department of Cardiology, Charité University Medicine Berlin, Berlin, Germany; ^4^Philips Healthcare Systems, Hamburg, Germany

**Keywords:** CMR, COVID-19, SARS-CoV-2 vaccination, myocarditis, pericarditis

## Abstract

Serious adverse events associated with new vaccines targeting SARS-CoV-2 are of high interest to the public and to public health as a worldwide mass immunization campaign has been initiated to contain the ongoing COVID-19 pandemic. We describe a series of 4 individuals with signs of a myocarditis/pericarditis according to cardiac MRI results in temporal association with currently in the European Union authorized SARS-CoV-2 vaccines. We found mild abnormal MRI results independent of the type of SARS-CoV-2 vaccine. There is a need of continuing monitoring outcomes of myocarditis cases after COVID-19 vaccination as recently published cases suggest an uncomplicated short-term course whereas the long-term implications are not yet known but taking the available evidence into account the benefits of using COVID-19 vaccines still clearly outweigh the risks.

## Background

Before the COVID-19 pandemic, there have only been a few reports of myocarditis and pericarditis as an adverse event following immunization with the exception of cases following live-attenuated smallpox vaccine (1, 2).

Serious adverse events associated with new vaccines targeting SARS-CoV-2 are of high interest to the public and to public health as a worldwide mass immunization campaign has been initiated to contain the ongoing COVID-19 pandemic.

SARS-CoV-2 vaccines currently authorized for use in the European Union by the European Pharmacy Agency include the messenger RNA (mRNA) vaccine Comirnaty (Pfizer-BioNTech), Spikevax (Moderna) and the vector-based vaccines Vaxzevria (AstraZeneca), and Vaccine Janssen (Johnson and Johnson) (3).

We describe a series of 4 individuals with signs of a myocarditis/pericarditis according to cardiac MRI results in temporal association with SARS-CoV-2 vaccination to investigate any differences regarding the phenotype.

## Materials and Methods

For this report we retrospectively reviewed cardiac MRI exams performed at our institution between 07/01/2021-09/06/2021 for MRI findings of cardiac inflammation such as myocarditis or pericarditis associated with SARS-CoV-2 Vaccination. We reviewed the medical records regarding the timing of COVID-19 or SARS-CoV-2 vaccination and the vaccine used. All available demographic, clinical or laboratory information were documented (Table 1).

**Table 1:** Four patients diagnosed with signs of myocarditis/pericarditis in temporal relation to a SARS-CoV-2 vaccination.

**Clinical data**	**Patient 1**	**Patient 2**	**Patient 3**	**Patient 4**
Demographic data	21 years old/male	42 years old/male	18 years old/male	18 years old/male
Type of vaccine	2nd dose Spikevax (Moderna)	2nd dose Comirnaty (Pfizer-BioNTech)	Janssen (Johnson and Johnson)	Vaxzevria (AstraZeneca)
Symptoms	Chest pain and discomfort, malaise, dyspnea, limited physical capacity	Chest pain and discomfort, dyspnea, limited physical capacity	Chest pain and discomfort, dyspnea, limited physical capacity	Chest pain and discomfort, limited physical capacity
Vaccination–symptoms (days)	1	2	1	12
Vaccination–Cardiac MRI (days)	6	8	57	68
Troponin (ng/ml)	Troponin-T-hs 526 (normal <14 ng/l)	Troponin-I-hs 4,868 (normal <34.1 ng/l)	NA	Troponin-I-hs <5.1 (normal <34.1 ng/l)
NT-pro-BNP (ng/l)	79 (normal <97 ng/l)	40 (normal <100 ng/l)	NA	NA
Coronary angiography	No pathological findings	No pathological findings	NA	No pathological findings
Pulmonary angiography	No pathological findings	NA	NA	NA
**Cardiac MRI results**				
LV-EF (%) (normal 57 to 77%)	58	64	60	60
RV-EF (%) (normal 52 to 72%)	59	NA	55	62
GLS (%) (normal −28.5 to −20.5%)	−21. 2	−17.8	−23.2	−24
ECV (%) (normal ≤ 30%)	21	25	26	25
Wall motion abnormalities	+	–	–	–
Local T2w signal abnormality	+	+	+	–
Elevated global T2 relaxation time	–	–	–	–
Elevated global T1 relaxation time	–	–	–	–
Pericardial effusion	–	+	+	+
Local LGE	+	–	+	–

*LV-EF, Left ventricular ejection fraction; RV-EF, Right ventricular ejection fraction; GLS, Global longitudinal strain; ECV, Extracellular volume; LGE, Late gadolinium enhancement; NA, Not available; NT-pro-BNP, N-terminal pro-B-type natriuretic peptide*.

The study complies with the declaration of Helsinki. Approval was obtained from the ethics committee of Charite – Unversitätsmedizin Berlin. All examinations have been clinically indicated.

Cardiac MRI was performed at 1.5T/3T [Philips Healthcare, Best, Netherlands] and evaluated using a standardized diagnostic protocol as described previously (4). The protocol included cine, T1 and T2 mapping, and late gadolinium enhancement (LGE) images. Cutoffs for elevated T1 values (normal 903 to 1,085 ms at 1.5 Tesla and 1,173 to 1,334 ms at 3 Tesla) and T2 values (normal 41 to 57 ms at 1.5 Tesla and 35 to 51 at 3 Tesla) were based on 2 standard deviations above the respective means in a healthy reference group examined on the same scanners. Clinical cardiac MRI reports were reviewed by three cardiologists experienced in cardiovascular imaging in consensus.

## Cases

### Patient 1

Patient 1, a healthy 21-year-old male, received his second vaccination dose of Spikevax (Moderna). The following day the patient complained about chest pain and discomfort, shortness of breath, limited physical capacity and malaise. At presentation to the hospital the electrocardiogram showed no pathological findings. The serum levels for C-reactive protein and NT-proBNP were normal. High-sensitive Troponin T was elevated up to 526 ng/l (normal <14 ng/l). A coronary angiography was performed with exclusion of a coronary artery disease. CT pulmonary angiography excluded a pulmonary embolism. Transthoracic echocardiography showed normal myocardial function without wall motion abnormalities or relevant valvular heart disease.

**Figure 1:**
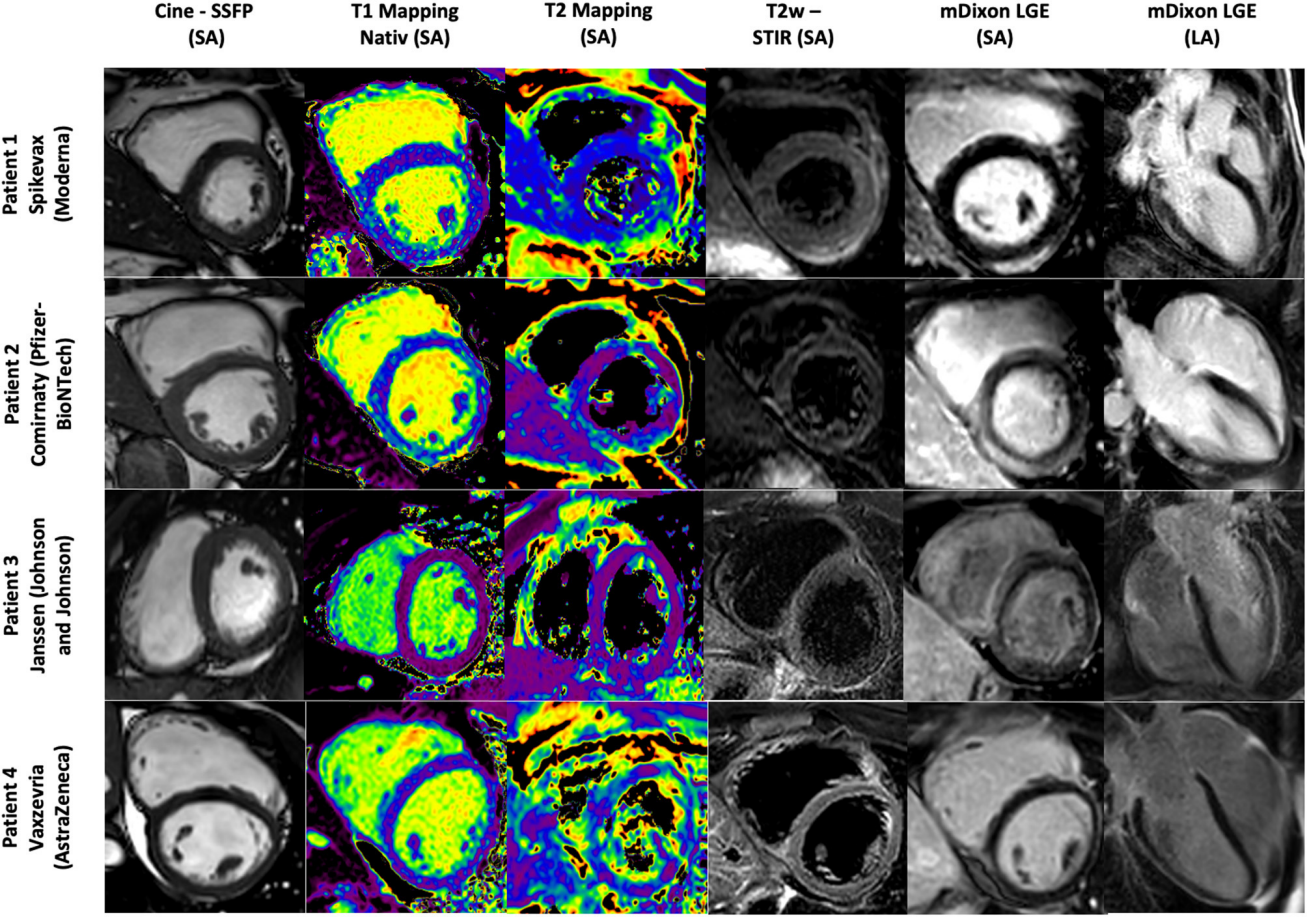
Cardiac MR images of Patient 1–4. Patient 1 with signs of a myopericarditis after mRNA SARS-CoV-2 vaccination with Spikevax (Moderna). Patient 2 with signs of a myocarditis after mRNA SARS-CoV-2 vaccination with Comirnaty (Pfizer-BioNTech). Patient 3 with signs of a discrete pericarditis in the LV-lateral area after SARS-CoV-2 vaccination with Janssen (Johnson and Johnson). Patient 4 with a mild to moderate pericardial effusion up to 11 mm in the medial posterior wall of the LV as a possible residual of an expired pericarditis/myocarditis after SARS-CoV-2 vaccination with Vaxzevria (AstraZeneca). SSFP, Stady state free precession; T2w FSE, T2-weighted fast spin echo; mDixon, Single breath-hold three-dimensional (3D) ECG-gated multi-echo chemical shift-based sequence; T1 relaxation were calculated from single breath-hold two-dimensional (2D) modified Look-Locker inversion recovery (MOLLI) sequence LA, long axis; SA, short axis.

Cardiac MRI at 3 Tesla showed a normal left and right ventricular size with normal left and right ventricular ejection fraction and normal values for the global longitudinal strain. T2 weighted images indicated a regional edema anterolateral/inferolateral (basal) with corresponding elevated quantitative myocardial T2 mapping parameters up to 70 ms (normal 35 to 51 ms at 3 Tesla) (Figure 1). Corresponding patchy subepicardial LGE indicating inflammatory myocardial necrosis (Figure 1). Pericardial enhancement in the LGE and T2 weighted images in corresponding locations indicated a pericardial involvement (Figure 1). The global T1 relaxation time (1,227 ms, normal 1,173 to 1,334 ms at 3 Tesla) and global T2 relaxation time (43 ms, normal 35 to 51 ms at 3 Tesla) were normal. The patient was discharged after 6 days with stable cardiopulmonary parameter and improved symptoms with an anti-inflammatory therapy with ibuprofen and a supportive therapy with ACE-inhibitor and outpatient follow-up appointments.

### Patient 2

Patient 2, a healthy 42-year-old male, received the second vaccination dose of Comirnaty (Pfizer-BioNTech). Two days later, the patient presented to the emergency room of a referring hospital with chest pain and discomfort, shortness of breath and a decreased physical capacity.

The electrocardiogram on admission showed no pathological findings. The serum levels for C-reactive protein (59 mg/l, normal <5.0 mg/l) and cardiac necrosis marker were elevated with a highsensitive Troponin-I level of 4,868 pg/ml (normal <34.1 pg/ml) and creatinin kinase of 581 U/l (normal 190 U/l). The levels for D-Dimer and BNP were normal. A coronary angiography was performed with exclusion of a coronary artery disease. CT pulmonary angiography excluded a pulmonary embolism. Transthoracic echocardiography showed normal myocardial function without wall motion abnormalities or relevant valvular heart disease.

Cardiac MRI at 3 Tesla showed a normal left and right ventricular size with normal left and right ventricular ejection fraction but reduced values for the global longitudinal strain with −17.8% (normal −28.5 to −20.5% according to local reference values). T2 weighted images indicated a regional edema inferior/inferolateral (basal) with corresponding elevated quantitative myocardial T2 mapping parameters up to 53 ms (normal 35 to 51 ms at 3 Tesla) and corresponding subepicardial LGE in this region (Figure 1). The global T1 relaxation time (989 ms, normal 903 to 1985 ms at 1.5 Tesla) was normal. In summary, this provided evidence for acute myocarditis without functional limitation. An anti-inflammatory therapy with ibuprofen was started. During hospitalization, the patient complained of left lower leg pain. Duplex sonography of the veins showed thrombosis of a collateral vein in the region of the posterior tibial artery. Compression therapy and oral anticoagulation were started. The patient was discharged after 6 days with improved symptoms with recommendation to continue the anti-inflammatory, compression and oral anticoagulation therapy.

### Patient 3

Patient number 3 was an 18-year-old healthy and athletic young man. The patient reported that shortly after a vaccination with Janssen (Johnson and Johnson), he initially experienced an episode of fever and limb pain. The initial symptoms subsided significantly after 3 days. However, a marked limitation of physical capacity and a feeling of chestpain and discomfort at rest and under physical stress remained. After the complaints persisted even for 2 months after vaccination, an outpatient presentation was made for further diagnostics. The electrocardiogram showed no pathological findings. Transthoracic echocardiography showed normal myocardial function without wall motion abnormalities or relevant valvular heart disease. Cardiac MRI at 1.5 Tesla showed a normal left and right ventricular size with normal left and right ventricular ejection fraction and normal values for global longitudinal strain. The images showed a mild pericardial effusion in the area of the free RV wall and the basal posterior LV wall up to a maximum of 4 mm with evidence of inflammatory changes of the pericardium in the T2 weighted images and the LGE images in the area of the lateral LV wall. The global T2 relaxation time (49 ms, normal 35 to 51 ms at 1.5 Tesla) and T1 relaxation time (1,071 ms, normal 903 to 1,085 ms at 1.5 Tesla) were normal. Assuming a discrete pericarditis of the LV-lateral area, a native MRI follow-up after 3 months was recommended.

### Patient 4

Patient number 4, a healthy 18-year-old male received the first dose of Vaxzevria (AstraZeneca) at the end of June 2021. The patient reported new episodes of chestpain and discomfort and exercise limitation ~10 days after the vaccination. Initially, no medical presentation was made in the expectation that the symptoms would disappear. After a clear increase of the symptoms in the course of time, the patient presented to the emergency department of a referring hospital. The electrocardiogram on admission showed ST segment elevation in the inferior leads (II, III, and aVF). A coronary angiography was performed with exclusion of a coronary artery disease. The serum levels for C-reactive Protein [<1.0 mg/l, normal < 5.0 mg/l)] and highsensitive Troponin-I [<5.1 pg/ml, normal <34.1 pg/ml)] were normal. Creatinin kinase was increased with a serum level of 255 U/l (normal <190 U/l). Transthoracic echocardiography showed normal myocardial function without wall motion abnormalities or relevant valvular heart disease.

Cardiac MRI at 1.5 Tesla showed a normal left and right ventricular size with normal left and right ventricular ejection fraction and normal values for global longitudinal strain. The examination showed a mild to moderate pericardial effusion up to 11 mm in the mid-posterior wall of the LV. There was no evidence of acute cardiac inflammation in the T2 weighted and LGE images. The global T2 relaxation time (52 ms, normal 35 to 51 ms at 1.5 Tesla) and T1 relaxation time (979 ms, normal 903 to 1,085 ms at 1.5 Tesla) were normal. In the absence of signs of acute cardiac inflammation in the T2 weighted images and the LGE images, the pericardial effusion was considered as a possible residual of an expired pericarditis/myocarditis.

## Discussion

Myocarditis is an inflammation of the heart muscle in the absence of ischemia (5). If it is accompanied by pericarditis, an inflammation of the pericardium, it is referred to as myopericarditis. Myocarditis is predominantly mediated by viral infection, but can also be induced by bacterial, protozoal or fungal infections as well as systemic immune-mediated diseases and a variety of toxic substances and certain drugs as well as vaccine exposures (5).

For vaccine associated myocarditis the underlying mechanisms are not fully understood either. Molecular mimicry between the spike protein of SARS-CoV-2 and self-antigens, trigger of preexisting dysregulated immune pathways, immune response to mRNA, and activation of immunologic pathways, and dysregulated cytokine expression have recently been proposed (6).

Vaccine associated myocarditis is still overall rare and more common in males and the young population (7). The Advisory Committee on Immunization Practices (ACIP) recently published an incidence of 40.6 cases per million second doses of mRNA SARS-CoV-2 vaccinations in a population of males aged 12–29 years compared to 2.4 per million second doses administered to males aged ≥30 years (7).

The reasons for male predominance is unknown, but theories relate to sex hormone differences in immune response and myocarditis and underdiagnosis of cardiac disease in women (6).

Severity and clinical presentation of myocarditis or pericarditis vary among patients. Symptoms might include dyspnea, chest pain or palpitations, although especially in younger children other symptoms might be present (8). The clinical diagnostic evaluation might show elevated cardiac injury marker, pathological findings on electrocardiogram, echocardiogram, or as shown cardiac MRI results.

As also seen in our patients the clinical course of SARS-Cov2 vaccination associated myocarditis is typically mild and self-limited (2). Data published by the Israeli Ministry of Health showed 148 cases of myocarditis among 10.4 million vaccinated Israelis (9). Most cases occurred within 30 days after the second dose of a mRNA vaccination. Most cases required a hospitalization up to 4 days but were considered mild (9).

Regarding the guidelines for a mild and uncomplicated myocarditis/pericarditis a myocardial biopsy or viral serology was not performed in our patients. According to the guidelines the management depends on supportive therapy with targeted cardiac and anti-inflammatory medications and specific interventions if necessary (10, 11). Exercise restriction is recommended until the heart recovers (10, 11).

We found mild abnormal MRI results independent of the type of SARS-CoV-2 vaccine. We hypothesize, that abnormal findings might be present independent of vaccine and potentially might also be present when patients are flu-vaccinated. Future research work should also focus on this aspect. A more pragmatic approach might be to look first for cardiac abnormalities in cases of cardiac symptoms after vaccination, such as elevated lab values as troponin and NTproBNP or abnormalities at echocardiography as previously described from our group post COVID-19 (12).

In conclusion clinicians should be aware of vaccine-induced myocarditis as a possible adverse event after SARS-CoV-2-vaccination.

There is a need of continuing monitoring outcomes of myocarditis cases after COVID-19 vaccination as recently published cases suggest an uncomplicated short-term course whereas the long-term implications are not yet known.

Taking into account the available evidence including the risks of myocarditis and pericarditis, it can be determined that the benefits of using COVID-19 vaccines still clearly outweigh the risks. There is a need for a continued educational campaign for the public regarding the risk of COVID-19 and the benefits and risks of a SARS-CoV-2 vaccination.

## Data Availability Statement

The original contributions presented in the study are included in the article/supplementary material, further inquiries can be directed to the corresponding author/s.

## Ethics Statement

The studies involving human participants were reviewed and approved by Charite Unversitätsmedizin Berlin. The patients/participants provided their written informed consent to participate in this study. Written informed consent was obtained from the individual(s) for the publication of any potentially identifiable images or data included in this article.

## Author Contributions

The first draft of the manuscript was written by CJ and all authors commented on previous versions of the manuscript. All authors contributed to the study conception and design, read, and approved the final manuscript.

## Funding

SK received funding from the DZHK (German Centre for Cardiovascular Research) and by the BMBF (German Ministry of Education and Research).

## Conflict of Interest

SK as a researcher is supported by a grant from Philips Healthcare. CS is an employee of Philips Healthcare. The following authors report financial activities outside the submitted work: BP reports having received consultancy and lecture honoraria from Bayer Daiichi Sankyo, MSD, Novartis, Sanofi-Aventis, Stealth Peptides and Vifor Pharma; and editor honoraria from the Journal of the American College of Cardiology. The remaining authors declare that the research was conducted in the absence of any commercial or financial relationships that could be construed as a potential conflict of interest.

## Publisher's Note

All claims expressed in this article are solely those of the authors and do not necessarily represent those of their affiliated organizations, or those of the publisher, the editors and the reviewers. Any product that may be evaluated in this article, or claim that may be made by its manufacturer, is not guaranteed or endorsed by the publisher.
